# Psychometric evaluation of the mandarin version of the caregiver task inventory (CTI-45) for family caregivers of stroke survivors

**DOI:** 10.1186/s40359-025-02970-z

**Published:** 2025-07-01

**Authors:** Qi Lu, Linda Johansson, Jan Mårtensson, Yue Zhao, Kristofer Årestedt

**Affiliations:** 1 Studies of integrated health and welfare (SIHW), School of Health and Welfare, Jönköping University, Jönköping, Sweden; 2 Intelligent Nursing Research Center, Tianjin Medical University, Tianjin, China; 3 School of Nursing, Tianjin Medical University, No.22 Qixiangtai Road, Heping District, Tianjin, 300070 China; 4 Institute of Gerontology, Studies of integrated health and welfare (SIHW), School of Health and Welfare, Jönköping University, Jönköping, Sweden; 5 Department of Nursing science, Studies of integrated health and welfare (SIHW), School of Health and Welfare, Jönköping University, Jönköping, Sweden; 6 Faculty of Health and Life Sciences, Linnaeus University, Kalmar, Sweden; 7 Department of Research, Region Kalmar County, Kalmar, Sweden

**Keywords:** Family caregivers, Measurements, Needs, Psychometrics, Stroke.

## Abstract

**Background:**

This study aimed to translate the Caregiver Task Inventory-45 (CTI-45) into Mandarin and evaluate its psychometric properties for use among family caregivers of stroke survivors in China.

**Methods:**

This methodological study included cognitive interviews and a psychometric evaluation of the CTI-45. Questionnaires were distributed to family caregivers at four tertiary hospitals in Tianjin, China. In addition to the eight family caregivers who participated in the cognitive interviews, 251 family caregivers of stroke survivors aged ≥ 18 years, who were biologically or legally related to the survivors, were providing unpaid home care, and were proficient in Mandarin, completed the survey. The CTI-45 was translated according to the European Organisation for Research and Treatment of Cancer (EORTC) translation protocol. Cognitive interviews were then conducted to evaluate the Mandarin CTI-45 using ordinal confirmatory factor analysis (CFA). The suggested one- and three-factor models were both evaluated. Internal consistency reliability was assessed using ordinal alpha and ordinal omega coefficients.

**Results:**

The suggested three-factor model was superior compared to the one-factor model and demonstrated acceptable model fit (RMSEA = 0.042, CFI = 0.996, TLI = 0.995, SRMR = 0.072). All three scales demonstrated excellent reliability according to ordinal alpha and ordinal omega: Direct care to the patient 0.96/0.92, Intrapersonal tasks 0.97/0.94, and Interpersonal ties 0.95/0.90.

**Conclusions:**

The Mandarin version of the CTI-45 demonstrated good psychometric properties and could be used to assess the multidimensional care needs of family caregivers caring for stroke survivors.

## Background

Strokes represent the second leading cause of death and the third leading cause of disability globally, posing a considerable burden on stroke survivors, their families, and society [1, 2]. In China, strokes are the leading cause of death and disability, primarily because of its aging population and the widespread prevalence of chronic diseases such as hypertension and diabetes [3]. Although medical treatment and healthcare have improved in recent years, leading to higher survival rates [4, 5], many stroke survivors nevertheless experience long-term disabilities and rely on others for help after leaving the hospital. Family caregivers play a crucial role in providing this type of support [6, 7].

After receiving in-hospital care, stroke survivors are typically discharged to their homes, rehabilitation care units, or long-term care facilities [8]. In China, where the influences of traditional filial piety remain strong and medical resources may be limited, most survivors return home once their condition stabilises and rely on family caregivers for the support they require [9]. Nevertheless, family members often find themselves unprepared for the task of caring for stroke survivors at home, which may result in feelings of caregiver burden [10].

A number of studies have revealed that the family caregivers of stroke survivors frequently have unmet needs [7, 11] For instance, one systematic review demonstrated that these caregivers lack sufficient skills, support, and information to effectively transition into their new roles and access appropriate services [7]. Furthermore, as survivors progress through rehabilitation and their caregivers become more experienced, their needs may evolve over time [12]. Therefore, structured assessments are necessary to identify the needs of caregivers during the rehabilitation process. Such assessments require self-reported instruments, as these offer direct insights into caregivers’ experiences, feelings, and challenges—which are essential for providing tailored support and enhancing care outcomes. Such instruments must possess satisfactory psychometric properties to accurately assess the perceived needs of family caregivers.

Several instruments have been developed to measure caregiver needs, such as the Caregiver Strain Index (CSI), Zarit Burden Interview (ZBI), and Caregiver Burden Inventory (CBI) [13, 14, 15]. However, these tools primarily assess stress or burden and indirectly reflect caregiver needs [16]. For example, the CSI identifies areas of strain and potential support needs, focusing on the impact of caregiving on daily life. The ZBI measures emotional, physical, and social burden. The CBI provides a comprehensive assessment of caregiving strain across five dimensions: time dependence, developmental, physical, social, and emotional burden. These tools are useful for identifying stress but lack direct assessment of broader needs for caregivers, such as practical support, emotional well-being, and long-term care planning. This gap underscores the necessity for instruments like the Caregiver Task Inventory (CTI-45), which explicitly targets multidimensional needs to inform tailored interventions for stroke caregivers.

The Caregiver Task Inventory (CTI-45) was developed in the United States to help family caregivers of stroke survivors improve their caregiving skills [17]. The original version comprises 45 caregiving-related items divided into three subscales: direct care for the patient, intrapersonal tasks, and interpersonal ties. While the CTI-45 was designed to identify caregivers’ multidimensional needs, its psychometric properties remained unexplored. A prior study by Chan and Chang (1999) tested the original CTI in a small sample of 29 Chinese family caregivers of terminally ill cancer patients, reporting a Cronbach’s alpha of 0.86 for the total scale and 0.67–0.81 for its subscales [18]. However, this limited evaluation of CTI in a narrow population underscores the need for rigorous psychometric examinations of its reliability and validity in broader Chinese contexts.

 In 2011, Lee and Mok developed a shortened Chinese version of the CTI, known as the CTI-25, considering cultural differences and the fact that the English version of the CTI-45 is quite extensive [19]. The CTI-25 includes five dimensions: learning to cope with a new role, providing care according to the care receiver’s needs, managing one’s own emotional needs, appraising supportive resources, and balancing caregiving needs with personal needs. However, a qualitative analysis of 26 interviews with stroke caregivers revealed that financial needs (e.g., item 27: “Assume financial costs, actual and potential”) are critical areas addressed by items removed from the CTI-45 [11]. Other important aspects like long-term care planning (e.g., Item 28: ‘Confront the possibility of institutionalization’) and familial role adjustments (e.g., Item 35: ‘Balance the giving of assistance with responsibilities to other family members’) are also covered in the CTI-45 but not in the CTI-25. These items are essential for capturing the complexity of caregiving in China. Additionally, the CTI-45 is not currently available in Mandarin—one of the primary languages spoken in China.

Thus, this study aimed to translate the CTI-45 into Mandarin and evaluate its psychometric properties for use among family caregivers of stroke survivors in China.

## Methods

### Study design

This methodological multicentre study was conducted in two stages: (1) translation and (2) psychometric evaluation. Permission to translate the CTI-45 into Mandarin and evaluate its psychometric properties was obtained from the instrument’s developer [16].

### Translation of the Mandarin version of CTI-45

The translation process followed the European Organization for Research and Treatment of Cancer’s translation protocol (4th Edition) [20]. A translation unit was formed, consisting of the authors of this report, with the first author (QL) serving as the translation coordinator.

Two independent translators, both native Chinese Mandarin speakers, undertook the task of translating the English version of the CTI-45 into Mandarin. Two native Chinese-speaking authors from the research team (QL and YZ) then carefully compared the two Mandarin translations, resolved any discrepancies, and produced a revised version. To ensure accuracy, two independent translators, both native English speakers, performed back-translations. These back-translated versions were then compared with the original version of the instrument, leading to minor revisions that produced a reconciled Mandarin version. Finally, an independent Chinese-Canadian linguist externally proofread the reconciled Mandarin version and compared it to the original English text. After thorough deliberation and consensus within the research team, the initial Mandarin version of the CTI-45 was finalised.

The initial Mandarin version of the CTI-45 was further refined using cognitive interviews. Eight unpaid caregivers and relatives of stroke survivors were selected to test the instrument. To gain insights into their thought processes, both thinking-aloud and verbal probing techniques were used—as advocated by Willis [21]. The face-to-face interviews were conducted in a private setting, and lasted between 60 and 90 min. Based on the feedback obtained from these interviews, minor modifications were made to items that were perplexing or challenging to comprehend. This involved addressing issues such as confusing words and ambiguous language. These revisions led to the development of the final Mandarin version of the CTI-45, which was then used for the psychometric evaluation.

### Psychometric testing of the Mandarin version of CTI-45

### Sample and procedure

Participants were recruited between January 2023 and May 2023 from four tertiary hospitals in Tianjin, China. The inclusion criteria for the study were: family caregivers of stroke survivors aged ≥ 18 years, biologically or legally related to the survivors, providing unpaid home care, and proficient in Mandarin. Any respondents who refused to participate were excluded. The sample size for this study was determined based on the number of items in the CTI-45. A sample size of 5 times the number of items, totalling 225 caregivers, was used [22]. Simulation studies have shown that 200 observations can be sufficient for CFA analyses, dependent on estimation methods [26].To account for the risk of missing data, 270 family caregivers were invited to participate.

### Measurements

Data were collected through a questionnaire encompassing demographic information and a wide range of self-reported items—including the Mandarin version of the CTI-45. Demographic information included gender, age, educational level, occupation, economic situation, relationship to the patient, role in caregiving, living together with the patient, prior caregiving experience, caregiving duration, daily caregiving hours, and severity of the patient’s condition (time for diagnose and number of stroke).

The Mandarin version of the CTI-45 replicates the structure of the original scale, comprising 45 items categorised into three specific subscales: direct patient care (items 1–14), intrapersonal tasks (items 15–33), and interpersonal ties (items 34–45). Participants responded to each item on a 3-point scale, where 0 indicated not difficult, 1 indicated difficult, and 2 indicated extremely difficult. The total score of the instrument ranged between 0 and 90, whereas the subscale scores ranged between 0 and 28 for direct patient care, 0–38 for interpersonal tasks, and 0–24 for interpersonal ties. Higher scores indicate greater perceived difficulty in managing caregiving tasks.

### Data collection

For this study, data were collected on-site at hospitals. Professionally trained nurses and graduate students were assigned to identify and contact eligible family caregivers of stroke survivors. Prior to data collection, the caregivers were fully informed regarding the research objectives, methods, and measures taken to safeguard the confidentiality of their personal information. The caregivers then completed the questionnaires independently, with additional assistance provided to those who encountered reading difficulties. Upon completion, the questionnaires were collected and data entry and verification tasks were assigned to two doctoral students to guarantee accuracy and data integrity.

### Data analysis

Descriptive statistics were used to present the participants’ demographic characteristics. Continuous variables were described using means and their associated standard deviations (SDs), whereas categorical data were represented as frequencies and percentages.

All items in the CTI-45 were treated as ordered categories. To describe the score distribution, frequencies, percentages, medians, quartiles, and/or skewness statistics were used.

The psychometric properties of the Mandarin version of the CTI-45 were evaluated in terms of construct validity (latent structure) and internal consistency reliability. Due to the ordinal nature of data, the latent structure of the CTI-45 was evaluated through ordinal confirmatory factor analysis (CFA) using polychoric correlations and weighted least-squares mean-variance (WLSMV) estimation. Initially, a proposed one-factor model was evaluated, followed by a three-factor model. The model fit was examined using the Standardised Root Mean Residual (SRMR), Root Mean-Square Error of Approximation (RMSEA), Comparative Fit Index (CFI), and Tucker-Lewis Index (TLI) [23–25]. Good model fit was defined as SRMR ≤ 0.08, RMSEA ≤ 0.06, CFI ≥ 0.95, and TLI ≥ 0.95, while reasonable fit was defined as RMSEA ≤ 0.08, CFI ≥ 0.90, and TLI ≥ 0.90. Further evaluation of the model fit considered the strength of factor loadings, and the absence of cross-loadings, correlated error variances, and Haywood cases [26]. The scaled chi-square difference test, based on the work of Satorra and Bentler was used to compare nested CFA models [33].

The reliability of the Mandarin CTI-45 was evaluated in terms of internal consistency, and was examined using ordinal alpha and ordinal omega. Following recent methodological recommendations [27], McDonald’s omega was also calculated. This approach complements ordinal alpha and ordinal omega to ensure comprehensive evaluation of internal consistency. Values > 0.70 were taken to suggest acceptable internal consistency [28, 29].

All analyses were conducted using R version 4.2.1 (R Foundation for Statistical Computing, Vienna, Austria), with the following packages: semTools 0.5-6, psych 2.3.6, tidyverse 1.3.1, sjmisc 2.8.9, summary tools 1.0.1, correlation 0.8.2, and lavaan 0.6.16.

## Results

### Sample characteristics

Of the 270 family caregivers originally invited to participate, 11 declined, resulting in a response rate of 96%. The reasons cited for not participating were primarily time constraints (*n* = 3), perceived length of the questionnaire (*n* = 5), and belief that the survey was not relevant to their specific caregiving situation (*n* = 3). Eight returned questionnaires were excluded from our analysis owing to certain missing CTI-45 data. As a result, our final sample consisted of 251 family caregivers who had completed all of the CTI-45 questions.

The sociodemographic characteristics of the family caregivers are summarised in Table 1. The final sample included 142 females (56.6%) and 109 males (43.4%) with a mean age of 52.1 years (SD = 13.5). Most were spouses or children of the stroke survivors (*n* = 202, 80.8%), and the majority cohabitated with them (*n* = 147, 58.6%). Most served as primary caregivers (*n* = 147, 58.8%) and had no prior caregiving experience (*n* = 141, 56.2%). A significant proportion had intermediate vocational education (*n* = 152, 60.5%), worked in skilled trade-based occupations (*n* = 159, 64.6%), and had average levels of economic security (*n* = 189, 75.3%).

**Table 1:** Sociodemographic characteristics (N = 251)

Variable	
Gender, N (%)	
Male	109 (43.4)
Female	142 (56.6)
Age, Mean (SD)	52.1 (13.5)
Age groups, n (%)	
≤ 40	57 (22.7)
41–60	121 (48.2)
61–80	70 (27.9)
>80	3 (1.2)
Education level, n (%)	
Low vocational education	20 (8.0)
Intermediate vocational education	152 (60.5)
High vocational education	79 (31.5)
Occupation, n (%)	
Blue collar (manual workers)	159 (64.6)
White collar (clerical workers)	87 (35.4)
Missing data	5
Economic situation, n (%)	
Extremely good	1 (0.4)
Good	12 (4.8)
Ordinary	189 (75.3)
Difficult	38 (15.1)
Extremely difficult	11 (4.4)
Relationship, n (%)	
Spouse	92 (36.8)
Children	110 (44.0)
Daughter/son-in-law	12 (4.8)
Parents	18 (7.2)
Others	18 (7.2)
Missing data	1
Role in caregiving, n (%)	
Primary caregiver	147 (58.8)
Not primary caregiver	28 (11.2)
Shared caregiving responsibilities	75 (30.0)
Missing data	1
Living together with the patient, n (%)	
Yes	147 (58.6)
No	104 (41.4)
Previous caregiving experience, n (%)	
Yes	110 (43.8)
No	141 (56.2)
Daily caregiving hours, n(%)	
More than 6 h	187 (74.5)
3 to 6 h	36 (14.3)
1 to 2 h	14 (5.6)
Less than 1 h	14 (5.6)
Caregiving duration, n(%)	
Less than 1 month	149 (59.6)
1–6 months	20 (8.0)
7–12 months	3 (1.2)
13–24 months	6 (2.4)
More than 24 months	72 (28.8)
Missing data	1
Time for diagnosis, n (%)	
< 1 month ago	139 (55.4)
1–6 months ago	11 (4.4)
7–12 months ago	9 (3.6)
13–24 months ago	11 (4.4)
> 24 months ago	81 (32.3)
Number of strokes, n (%)	
1	151 (60.2)
2	56 (22.3)
3 or more	44 (17.5)

### Item and scale score statistics

The item score distributions are presented in Table 2. All three response options were used for the items in the CTI-45, but the distributions for all items exhibited positive skewness and pronounced floor effects. Between 46.2% (item 27) and 83.7% (item 35) of the family caregivers reported no need for additional supports (i.e., “Not difficult”).

**Table 2:** Item statistics of the Mandarin version of caregiver task inventory (CTI-45) (N = 251)

		Score distribution, %			
Items		Not difficult	difficult	Extremely difficult	Skewness
Direct care to the patient					
1	Be available when needed	59.4	31.9	8.8	0.96
2	Supervise prescribed treatments and general recommendations	74.9	20.7	4.4	1.67
3	Evaluate options for treatment and/or service	59.4	34.3	6.4	0.94
4	Monitor course of condition and evaluate significance of changes	57.0	35.9	7.2	0.86
5	Evaluate strength/resources of the care-receiver	62.2	31.9	6.0	1.04
6	Anticipate needs for future assistance	51.4	39.8	8.8	0.69
7	Provide structure for care-receiver’s daily activities	72.1	22.7	5.2	1.52
8	‘Run interference’ for care-receiver in social and community settings	70.9	25.9	3.2	1.37
9	Normalize care-receiver routine, within bounds of the impairment	71.7	23.1	5.2	1.50
10	Supervise/directly manage care-receiver’s resources	66.5	29.5	4.0	1.18
11	Cope with upsetting behavior of the care-receiver	69.3	25.9	4.8	1.35
12	Maintain adequate communication with the care-receiver	78.5	17.5	4.0	1.93
13	Perform basic ADL for the care-receiver	77.3	19.5	3.2	1.80
14	Satisfy need for creativity/originality to offset tedious routines	63.8	33.5	2.8	0.96
Intrapersonal tasks					
15	Compensate for emotional drain from constant responsibility	61.0	33.9	5.2	0.97
16	Compensate for or recover personal time	53.0	37.5	9.6	0.75
17	Gain knowledge about the disease	59.8	27.9	12.4	0.96
18	Avoid severe drain on physical strength/health	51.8	41.8	6.4	0.65
19	Resolve guilt over ‘negative feelings’ towards care-receiver	73.3	23.9	2.8	1.49
20	Resolve feelings of disappointment or guilt over one’s performance	73.3	24.7	2.0	1.42
21	Make up for or avoid loss/restrictions on future plans and perspectives	57.4	37.5	5.2	0.82
22	Readjust personal routine	65.3	29.1	5.6	1.18
23	Compensate for disruption of sleep	55.8	33.1	11.2	0.84
24	Emotionally accept the likelihood of progressive downward course	52.6	34.7	12.8	0.73
25	Work through changes in the lifelong relationship between caregiver and care-receiver	69.7	25.1	5.2	1.39
26	Find a locus of blame for the condition/disease	49.4	38.7	12.0	0.64
27	Assume financial costs (actual and potential)	46.2	37.9	15.9	0.53
28	Confront the possibility of institutionalization	58.2	29.5	12.4	0.91
29	Compensate for or avoid loss/reduction of physical and emotional intimacy	67.3	27.1	5.6	1.27
30	Separate feelings regarding condition from feelings toward the care-receiver	72.1	24.7	3.2	1.44
31	Resolve uncertainty about one’s skills as a caregiver	59.4	35.5	5.2	0.90
32	Release tensions/feelings toward the care-receiver	70.1	27.9	2.0	1.22
33	Adjust to/cope with an uncertain future	61.0	35.9	3.2	0.86
Interpersonal ties					
34	Designate other responsible caregiver(s)	53.0	35.5	11.6	0.75
35	Maintain family communication and exchange of information	83.7	12.0	4.4	2.40
36	Balance the giving of assistance with responsibilities to other family members	74.1	21.9	4.0	1.61
37	Cope with the loss/restriction of future family plans	62.2	33.9	4.0	0.97
38	Manage feelings toward other family members who do not regularly help	75.3	19.5	5.2	1.71
39	Maintain the family as effective decision-making group over a long period of time	74.9	21.5	3.6	1.65
40	Give appropriate consideration to care-receiver’s options and preferences	78.1	19.5	2.4	1.81
41	Consider as a family the need for institutionalization	62.2	26.7	11.2	1.06
42	Interact with medical, health and social service professionals	71.3	25.5	3.2	1.40
43	Maintain knowledge of the service system and options	63.4	31.5	5.2	1.07
44	Act as advocate or third-party negotiator for the care-receiver	65.7	27.1	7.2	1.22
45	Maintain knowledge of reimbursement mechanisms	48.6	33.9	17.5	0.58

The median (IQR) of the total score of the Mandarin version of the CTI-45 was 16 (IQR: 4, 30). Regarding the three dimensions, the specific results were as follows: direct care for the patient had a median of three (IQR: 1, 9), interpersonal tasks had a median of seven (IQR: 2, 15), and interpersonal ties had a median of three (IQR: 1, 7). Higher scores indicated greater perceived difficulty in managing caregiving tasks.

### Factor structure

The evaluation of the one-factor baseline CFA model showed a good model fit according to CFI (0.986) and TLI (0.985), reasonable fit according to RMSEA (0.074), and bad fit according to SRMR (0.087), and the chi-square goodness of fit test (χ²(945) = 2235.58, *p* < 0.001) (Table 3). The factor loadings varied between 0.55 (item 17) and 0.87 (items 19 and 36), and were all significant, at *p* < 0.001. No Haywood cases were detected, but inspection of the standardised residual covariance matrix and modification index showed extensive issues, with correlated error variances between 11 item pairs (2–3, 3–4, 11–12, 19–20, 28–41, 29–30, 36–38, 42–43, 43–44, 43–45, 44–45).

**Table 3:** Goodness-of-fit indices for the confirmatory factor analyses models of the Mandarin version of CTI-45

	χ² goodness-of-fit				RMSEA					
	χ²	df	p-value		RMSEA	90%CI	p-Value	CFI	TLI	SRMR
Baseline one-factor model	2235.58	945	< 0.001		0.074	0.070–0.078	< 0.001	0.986	0.985	0.087
Baseline three-factor model	1595.33	942	< 0.001		0.053	0.048–0.057	< 0.001	0.993	0.992	0.076
Modified three-factor model	1344.33	938	< 0.001		0.042	0.037–0.047	< 0.001	0.996	0.995	0.072

Abbreviations: CFI, Comparative Fit Index; RMSEA, Root Mean Square Error of Approximation; SRMR, Standardised Root Mean Square Residual; TLI, Tucker-Lewis Index

As correlated error variances may indicate multidimensionality, the hypothesised three-factor model was evaluated. The baseline three-factor model exhibited a good fit across all indices without any modifications: χ²(942) = 1595.33, *p* < 0.001), RMSEA = 0.053, CFI = 0.993, TLI = 0.992, and SRMR = 0.076 (Table 3). The factor loadings ranged from 0.68 (item 1) to 0.87 (item 9) for direct patient care, from 0.65 (item 24) to 0.89 (item 19) for intrapersonal tasks, and from 0.62 (item 45) to 0.91 (item 36) for interpersonal ties. All factor loadings were statistically significant (*p* < 0.001). No Haywood cases were identified. Nevertheless, examination of the standardised residual covariance matrix and modification index still revealed issues with correlated error variance, particularly observed between items 2–3, 19–20, 28–41, and 43–44.

Items with correlated error variances were scrutinised, and the observed issues were explained by overlapping item content. Consequently, a modified three-factor model was evaluated, which allowed the error variances to correlate with one another. The model exhibited improved fit across all indices: RMSEA = 0.042, CFI = 0.996, TLI = 0.995, SRMR = 0.072, and χ²(938) = 1344.33, p < 0.001 (Table 3). The chi-square difference test indicated significant improvement over the baseline three-factor model (Δχ²(4) = 111.22, *p* < 0.001), though differences in approximate fit indices were minimal: ΔRMSEA = − 0.011, ΔCFI = 0.003, ΔTLI = 0.003, and ΔSRMR = − 0.004. The factor loading ranged between 0.68 (item 1) and 0.87 (item 9) for direct patient care, 0.57 (item 17) and 0.87 (item 29 and 30) for intrapersonal tasks, and 0.62 (item 45) and 0.91 (item 36) for interpersonal ties. All factor loadings were significant, at *p* < 0.001 (Table 4). The factor correlations were 0.91 for direct patient care and intrapersonal tasks, 0.89 for direct patient care and interpersonal ties, and 0.94 for intrapersonal tasks and interpersonal ties. No Haywood cases were identified using the modified three-factor model.

**Table 4:** Factor loadings, error variances, and reliability estimates for the modified three-factor model of the Mandarin version of CTI-45

Item	Direct care to the patient	Intrapersonal tasks	Interpersonal ties
Direct care to the patient			
1	0.68 (0.540)		
2	0.75 (0.436)		
3	0.81 (0.337)		
4	0.79 (0.383)		
5	0.83 (0.308)		
6	0.82 (0.333)		
7	0.76 (0.430)		
8	0.81 (0.337)		
9	0.87 (0.248)		
10	0.85 (0.278)		
11	0.81 (0.337)		
12	0.72 (0.475)		
13	0.78 (0.388)		
14	0.86 (0.261)		
Intrapersonal tasks			
15		0.85 (0.272)	
16		0.85 (0.276)	
17		0.57 (0.679)	
18		0.86 (0.259)	
19		0.84 (0.298)	
20		0.76 (0.420)	
21		0.79 (0.383)	
22		0.79 (0.373)	
23		0.70 (0.507)	
24		0.65 (0.580)	
25		0.82 (0.326)	
26		0.75 (0.445)	
27		0.73 (0.467)	
28		0.73 (0.468)	
29		0.87 (0.240)	
30		0.87 (0.250)	
31		0.80 (0.360)	
32		0.85 (0.284)	
33		0.83 (0.317)	
Interpersonal ties			
34			0.72 (0.480)
35			0.82 (0.336)
36			0.91 (0.317)
37			0.88 (0.220)
38			0.88 (0.222)
39			0.86 (0.264)
40			0.85 (0.271)
41			0.72 (0.477)
42			0.80 (0.367)
43			0.75 (0.437)
44			0.72 (0.478)
45			0.62 (0.617)
Reliability			
Ordinal alpha	0.96	0.97	0.95
Ordinal omega	0.92	0.94	0.90
McDonald’s omega	0.92	0.94	0.90

### Internal consistency reliability

The Mandarin version of the CTI-45 showed high internal consistency reliability. The ordinal alpha of the total scale was 0.98, and the corresponding values were 0.96, 0.97, and 0.95 for the subscales of direct patient care, intrapersonal tasks, and interpersonal ties, respectively.

The ordinal omega of the one-factor model (i.e. total scale) was 0.97. For the modified three-factor model (i.e. three subscales), the ordinal omega values were 0.92, 0.94, and 0.90 for direct patient care, intrapersonal tasks, and interpersonal ties, respectively.

The McDonald’s omega of the total scale was 0.97, and the corresponding values were 0.92, 0.94, and 0.90 for the subscales of direct patient care, intrapersonal tasks, and interpersonal ties, respectively.

## Discussion

To the best of our knowledge, this is the first study to evaluate the CTI-45 using robust statistical methods specifically tailored to ordinal data. Overall, the findings firmly support the hypothesised latent structure that encompasses the three interrelated subscales. Nevertheless, it was revealed that there were issues related to correlated error variances, and the estimated internal consistency reliability raised concerns regarding potential redundancy.

The Mandarin version of the CTI-45 exhibited minimal missing data, indicating that the items were easily comprehensible and considered meaningful by the family caregivers. This conclusion was reinforced by cognitive interviews conducted with the caregivers, which supported the content validity of CTI-45. Only eight participants failed to complete all of the CTI-45 items, primarily owing to its length and complexity, with missing data concentrated toward the end. While the CTI-25 is a shorter instrument, it may not capture all crucial aspects pertinent to family caregivers, such as financial needs, owing to the removal of certain items from the original CTI-45 [11] By contrast, the CTI-45 offers a more precise and comprehensive evaluation of caregivers’ needs, providing deeper insights into the challenges they face. Given its comprehensive nature, the CTI-45 is well-suited for clinical applications that require tailored multidimensional interventions.

All of the CTI-45 items exhibited positive skewness, and floor effects were common. More than half of the family caregivers reported no need for support. This trend was likely because these participants were new to their caregiving roles and lacked sufficient experience. Although all response options of the 3-point Likert scale were utilised, the score distribution was skewed toward non-existent needs, posing challenges to the sensitivity and responsiveness of the tool. Consequently, it is advisable not to administer the CTI-45 prematurely, particularly during the early stages of caregiving. This will help to ensure the accuracy of the assessment’s results; as a result, users can better understand and support the actual needs of family caregivers.

The hypothesised three-factor model proposed by Clark [17] was confirmed through CFA, despite several notable issues. Although the one-factor model fit appeared satisfactory based on indices such as CFI and TLI, RMSEA and SRMR suggested a misfit. Typically, RMSEA is regarded as a more conservative and robust fit index, whereas CFI and TLI are regarded as more liberal fit indices [26] These misfits may be attributable to correlated error variances, indicating the potential multidimensionality or redundant overlapping of items. Although this issue was partially addressed in the modified three-factor model, the correlated error variances persisted. Further examination identified four redundant pairs of items. Consequently, based on theoretical justifications, correlated error variances were allowed for these item pairs. For example, items 2 and 3 dealt with the supervision and evaluation of treatment options, whereas items 19 and 20 addressed feelings of guilt and disappointment towards care receivers. Similarly, items 28 and 41 concerned institutionalisation, and items 43 and 44 focused on knowledge of service systems and options. Although the revised three-factor model is supported by goodness-of-fit indices, it is crucial to acknowledge that local dependency can compromise instrument reliability. Future revisions of the CTI-45 should address this limitation.

The Mandarin version of the CTI-45 has an ordinal alpha of ≥ 0.95. Psychometric literature often suggests that alpha values exceeding 0.90 or 0.95 may indicate redundancy [28] However, other sources argue that alpha values > 0.90 are crucial for instruments designed to facilitate clinical decision-making at an individual level [30] Ordinal alpha typically yields higher values than traditional Cronbach’s alpha [26]. Therefore, for future revisions, it may be sufficient to streamline the instrument by eliminating redundant items, thereby enhancing its clinical utility while maintaining psychometric integrity.

This study had several notable strengths. The translation process adhered to the rigorous EORTC protocol, ensuring accuracy and reliability. The cognitive interviews improved comprehension and cultural adaptation. Additionally, the study accounted for the ordinal nature of the data using appropriate techniques such as ordinal CFA, ordinal alpha, and ordinal omega, in our analyses concerning latent structure and internal consistency [31].

However, this study had several limitations that merit acknowledgement. First, the sample size was determined based on a rule of thumb, resulting in a calculated sample size of 248. The recommended minimum sample size for conducting CFA using the WLSMV approach is 200 participants [32] While other sources suggest a range of 200–300 participants for simpler models, larger sample sizes are recommended for more complex models. Despite adhering to these recommendations, the attained sample size remained at the lower end of the suggested spectrum, potentially affecting the robustness of the analysis and the generalisability of the findings. Moreover, the length of the questionnaire may have posed a challenge to a few of the participants, potentially compromising the quality of the CTI-45. To mitigate this issue, we deliberately placed the CTI-45 at the beginning of the questionnaire, allowing participants to prioritise and complete it with fresher focus.

## Conclusions

This study describes evidence supporting the validity of the Mandarin version of the CTI-45, confirming its satisfactory validity and reliability for assessing the multidimensional needs of caregivers. In particular, it exhibited robust psychometric properties for assessing family caregivers of stroke survivors. Nevertheless, its reliability might be further strengthened by addressing issues such as overlapping items and correlated error variances. Future revisions may involve excluding specific items to enhance the psychometric qualities of the tool. Despite these considerations, the Mandarin version of the CTI-45 remains a valuable tool for researchers to precisely assess caregivers’ needs, thereby promoting high-quality care.

## Data Availability

The datasets used and/or analyzed in this study are available from the corresponding author upon reasonable request. Requests for data access will be considered based on the purpose and feasibility of the intended use, and in accordance with ethical and confidentiality considerations.

## Abbreviations

CFA: Confirmatory Factor Analysis
CFI: Comparative Fit Index
CTI-45: Caregiver Task Inventory-45
EORTC: European Organisation for Research and Treatment of Cancer
RMSEA: Root-Mean-Square-Error of Approximation
SRMR: Standardised Root Mean Residual
TLI: Tucker-Lewis Index
WLSMV: Weighted Least-Squares Mean-Variance
